# Sodergren Hemorrhoidal Symptom Severity Score as a Guide for Treatment of Hemorrhoidal Disease: A Cross-Sectional Study

**DOI:** 10.7759/cureus.89528

**Published:** 2025-08-07

**Authors:** Siddhant Govekar, Navin Kumar

**Affiliations:** 1 General Surgery, All India Institute of Medical Sciences, Rishikesh, Rishikesh, IND

**Keywords:** hemorrhoidal disease, hemorrhoids, patient-reported outcome measures for hemorrhoids, patient-reported severity assessment tool for hemorrhoids, sodergren score

## Abstract

Background and objectives

Hemorrhoidal disease (HD) is a common anorectal condition affecting a large number of adults worldwide. Lack of standardized outcomes limits treatment decisions in HD. Patient-reported outcome measures (PROMs), directly reported by the patients, offer standardized, patient-centric measures, aiding in HD severity assessment and treatment decisions. The Sodergren score is one such PROM that has shown significant predictive ability for surgical decision-making in HD patients. The Aim of this study was to find a Sodergren hemorrhoidal symptom severity cut-off score to predict the need for surgical management in hemorrhoidal disease.

Methodology

All patients > 18 years of age diagnosed with HD presenting to the outpatient clinics in the Colorectal Unit, who gave their consent to participate, were included in the study. Patients with anal fistula, anal fissure, inflammatory bowel disease (IBD), colorectal malignancy, prior hemorrhoidal surgery, on antiplatelet therapy, and pregnancy were excluded. The sample size calculated was 34. Based on the treatment, the patients were divided into non-surgical (A) and surgical (B) groups (17 in each group). The patients were made to fill out the Sodergren hemorrhoidal symptom severity score questionnaire. The final Sodergren score was recorded for data analysis.

Results

Sodergren score was evaluated in 34 HD patients; 17 in each treatment group. A significant difference in Sodergren scores was found between the non-surgical group (median: 4; interquartile range (IQR) 0-4 ) and the surgical treatment group (median: 7 (IQR 4-10) (P=0.001). The Sodergren score demonstrated an area under the receiver operating characteristic curve (AUC) of 0.834 (95% CI: 0.702 - 0.966) for predicting surgical decision-making, with an optimal cut-off score of ≥7 (sensitivity:58.8; specificity:88.2).

Conclusion

Sodergren score demonstrated utility as a patient-reported severity assessment tool to guide treatment outcomes in HD patients. A cut-off score of ≥7 was predictive of surgery.

## Introduction

Hemorrhoidal disease (HD) affects millions of people around the world and significantly alters the quality of life of a substantial number of working-age individuals [1]. The widely used clinical grading of hemorrhoids is based on Goligher’s classification [2]. There is no uniformity in the treatment of HD. The treatment is not just based on the type and severity of hemorrhoids, but also on the patient’s symptomatology and preference, and the expertise of physicians [3].

The results of clinical examination do not necessarily correlate with the patient’s symptoms, and the eventual clinical decision for the type of treatment depends on both the symptoms experienced as well as the examination findings [4]. The absence of standardized outcomes is one major limitation in deciding ideal treatment therapy for HD [5].

Nowadays, the patient’s quality of life has been integrated in determining the treatment outcome. But this did not enable comparison between treatment modalities. To overcome this, various patient-related outcome measures (PROMs) have been developed, which is a report of the status of a patient’s health condition that come directly from the patient, without interpretation of the patient’s response by a clinician or anyone else [6,7].

PROMs involving an appropriate symptom severity scoring system can be used as an alternative to measure the severity of HD, and thus can aid treatment decision making and allow for meaningful comparison of treatment outcomes [8]. Pucher et. al. developed and validated one such PROM for HD, the Sodergren score, which showed the ability to predict clinical decision making with operative versus ambulatory cases [9].

The Sodergren hemorrhoidal symptom severity score has two prerequisites. First, the possibility of any other pathology in the patient must be excluded, and second, there should be rectal bleeding. It consists of questions on the severity of symptoms of itching, pain at rest, pain on defecation, and on frequency of prolapse. A weighted system is employed for the Likert scale response to each question. The final output is a quantitative data type having a minimum possible value of 0 and a maximum possible value of 14. Pucher et. al. demonstrated a significantly higher score for patients who underwent excisional hemorrhoidectomy than those who were given ambulatory or no treatment (7.7 ± 3.9 vs. 2.8 ± 3.5) with an AUC (area under receiver operating characteristic curve) of 0.842 (95% CI 0.714-0.970), which was satisfactory [9]. However, they did not propose a cut-off point of the Sodergren score that could predict the need for surgery [9]. Sha et al. externally validated the Sodergren score and proposed that patients with a pre-treatment Sodergren score of 6 or more be considered for upfront surgery [10].

The aim of the study was to find a Sodergren hemorrhoidal symptom severity cut-off score to predict the need for surgical management in hemorrhoidal disease. The primary objective was to find the Sodergren score between the non-surgical and surgical treatment groups.

## Materials and methods

The present study was a prospective observational study conducted in the Colorectal Unit of the Department of General Surgery at a tertiary healthcare center in North India. The study received clearance from the Institutional Ethics Committee, All India Institute of Medical Sciences, Rishikesh (Approval No. AlIMS/IEC/22/254, dated May 27, 2022). Study participants included anyone above 18 years of age diagnosed with HD who gave their consent to participate in the study. Patients with anal fistula, anal fissure, inflammatory bowel disease (IBD), colorectal malignancy, prior hemorrhoidal surgery, spinal injury, on antiplatelet therapy, or pregnancy were excluded from the study. A written informed consent was obtained from all the participants of the study.

The sample size was calculated using MedCalc® Statistical Software, version 19.7.4 (MedCalc Software Ltd, Ostend, Belgium) [11], assuming an AUC of 0.735, a null hypothesis value of 0.5, α=0.05, power 80%, and a 1:1 allocation ratio. The estimated minimum sample size was 34 subjects (17 in each group). Based upon the treatment, the patients were categorized into two groups (17 in each group): non-surgical management (no treatment/conservative therapy/rubber band ligation/sclerotherapy) (group A) and surgical treatment (group B) (hemorrhoidectomy). Baseline demographic data and detailed history, including symptoms and disease characteristics, were documented.

The clinical grading of hemorrhoids was based on Goligher’s classification [2]. Patients then filled out the Sodergren hemorrhoidal symptom severity score questionnaire (Table 1). All components of the questionnaire were explained to participants prior to completion. Any queries raised were addressed by the principal investigator to ensure clarity. Simultaneously, they were independently evaluated by the clinician for an appropriate treatment decision (conservative or surgical). The principal investigator was blinded to the clinician’s decision during questionnaire administration, and the clinician was blinded to the Sodergren score while evaluating the patient. The final Sodergren score and responses, along with the treatment advised, were recorded in a Microsoft Excel 2019 (Version 16.0) (Microsoft Corp., Redmond, WA) sheet for data analysis.

**Table 1:** Sodergren hemorrhoid symptom severity scoring system Pucher et al. [9]

Symptom			Points scored
How often do you feel that you might have a lump at your anus (prolapse)?	0	No symptoms	0
	1	Mild/do not really bother me	0
	2	-	0
	3	Moderately bothersome	0
	4	-	4
	5	Severe	4
How severe are your symptoms of pain or discomfort at rest? (circle number from 1-5)	0	No symptoms	0
	1	Mild/do not really bother me	0
	2	-	0
	3	Moderately bothersome	3
	4	-	3
	5	Severe	3
How severe are your symptoms of pain /discomfort on opening your bowels? (circle number from 1-5)	0	No symptoms	0
	1	Mild/do not really bother me	0
	2	-	0
	3	Moderately bothersome	0
	4	-	3
	5	Severe	3
How often do you feel that you might have a lump at your anus (prolapse)?	0	Never	0
	1	Less than once a month	0
	2	More than once a month	0
	3	More than once a week	0
	4	Everyday	4

Statistical analysis

Baseline demographics, symptom characteristics (type and duration), disease characteristics (number and grade of hemorrhoids), Sodergren questionnaire responses, final Sodergren score, and treatment group were recorded in a Microsoft Excel 2019 (Version 16.0) spreadsheet. Statistical analysis was performed using IBM SPSS Statistics for Windows, version 29 (IBM Corp., Armonk, NY). Continuous variables were described using mean ± standard deviation or median with interquartile range. Categorical variables were described using frequency and proportion. Sodergren scores were compared between the non-surgical (group A) and surgical (group B) groups. The Shapiro-Wilk test and Q-Q plots assessed normality. Normally distributed continuous variables were analyzed with an independent t-test, while non-normally distributed variables were analyzed with the Mann-Whitney U test. Statistical significance was set at p < 0.05.

The AUC assessed the Sodergren score's effectiveness in surgical decision-making. Sensitivity, specificity, and predictive values determined the cut-off score, with the Youden Index identifying the optimal threshold. The Hosmer-Lemeshow test evaluated model fit, with p < 0.05 indicating good calibration.

## Results

In the surgical arm (group B), a total of 19 patients were screened before achieving the sample size of 17 (89.4%). One (5.2%) patient was excluded because of colorectal malignancy, and 1(5.2%) because of a spinal injury that impaired the ability of patient to sense pain around the anal region. The patient characteristics have been represented in Table 2.

**Table 2:** Patient characteristics Group A: Non-surgical treatment; Group B: Surgical treatment

Category	Total (n=34)	Group A (n=17)	Group B (n=17)	P-value
Male	27 (79.4%)	16 (94.1%)	11 (64.7%)	0.85
Female	7 (20.6%)	1 (5.9%)	6 (35.3%)	
Age (years), mean (SD)	45.9 (18.2)	41.5 (18.5)	50.3 (17.3)	0.163
Duration of symptoms (months), median (interquartile range)	24 (2-60)	2 (8 days-18 months)	60 (24-72)	<0.001
Grade I	6 (17.6%)	6 (35.3%)	0 (0%)	<0.001
Grade II	11 (32.4%)	9 (52.9%)	2 (11.8%)	
Grade III	13 (38.2%)	2 (11.8%)	11 (64.7%)	
Grade IV	4 (11.8%)	0 (0%)	4 (23.5%)	

No significant difference was found in the sex and age of the patients in Groups A and B. However, a significant difference was found in the duration of the symptoms in the two groups, with the non-surgical group having a median duration of two months (interquartile range (IQR) 8 days-18 months), and the surgical group having a median duration of 60 months (IQR 24-72 months) (p=0.001). A higher grade of hemorrhoids was associated with patients from the surgical group (p=0.001).

**Figure 1:**
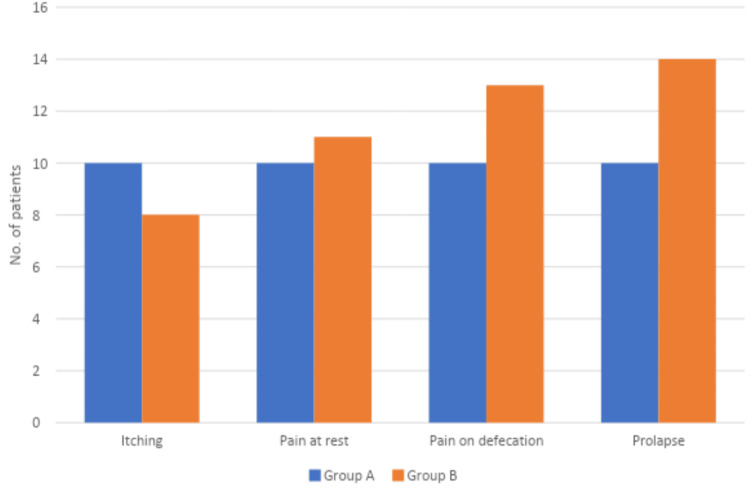
Frequency with which each of the questionnaire symptoms were reported Group A: Non-surgical treatment; Group B: Surgical treatment

The frequencies of the individual questionnaire symptoms reported by the patients have been shown in Figure 1. In the non-surgical group (group A), all symptoms (itching, pain at rest, pain on defecation, and prolapse) were seen with equal frequencies (10 (58.8%) patients each). In the surgical group (group B), the most commonly reported symptom was prolapse (14 (82.3%) patients), followed by pain on defecation (13 (76.4%) patients). Pain on defecation showed the highest severity on the Likert scale, with 8 (47.1%) patients reporting moderate to severe pain in the non-surgical arm and 11 (64.7%) patients reporting severe pain in the surgical arm. 6 (35.3%) patients reported prolapse every day in the non-surgical arm as compared to 13 (76.4%) in the surgical arm.

**Figure 2:**
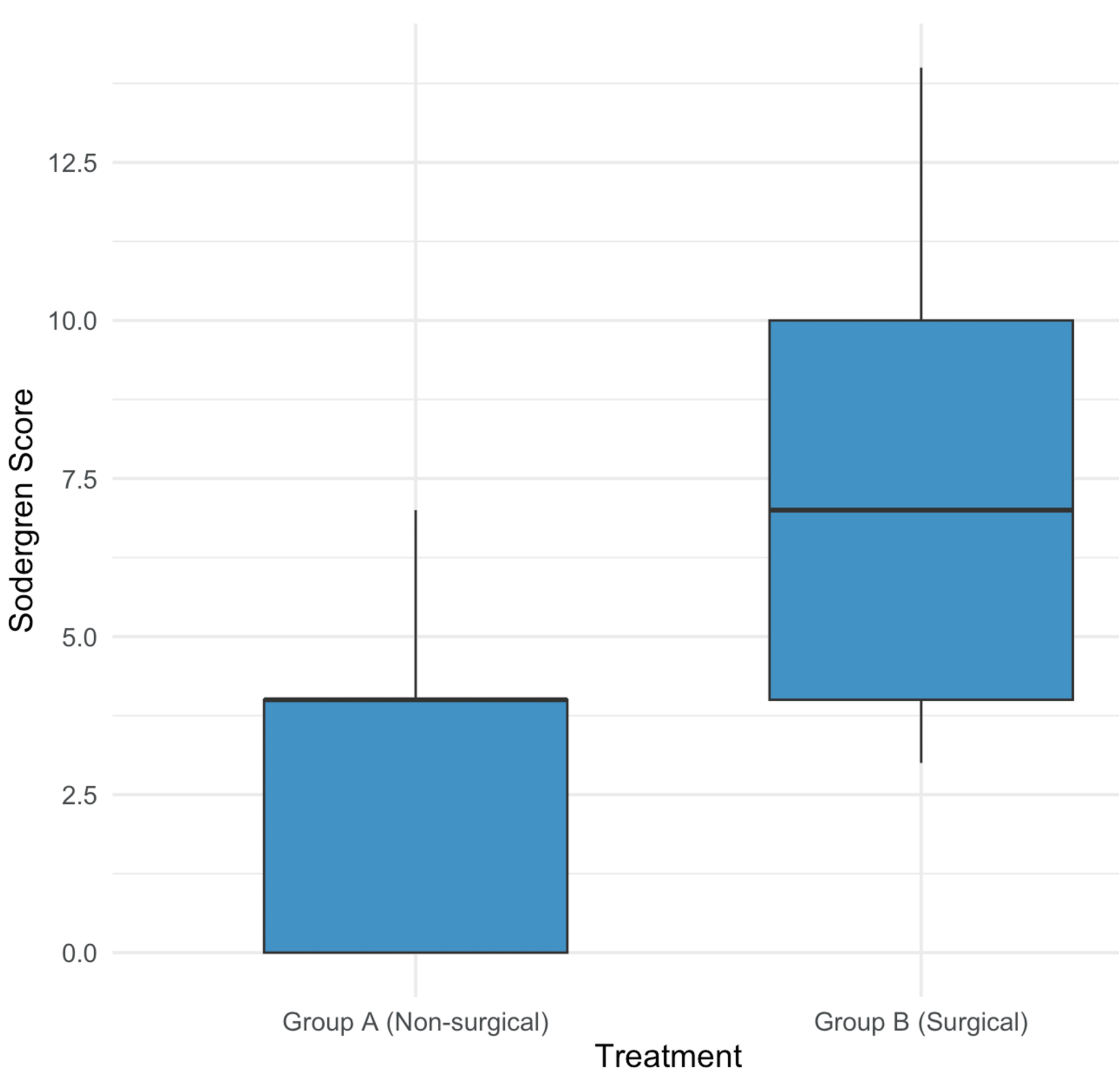
Box plot for Sodergren scores of the subjects (p=0.001) Group A: Non-surgical treatment; Group B: Surgical treatment

A statistically significant difference in the Sodergren scores of the two groups was found. Group A had a median score of 4 (IQR 0-4), while Group B had a median score of 7 (IQR 4-10) (p=0.001) (Mann-Whitney U test). The box plot diagram for subjects in each group has been illustrated in Figure 2.

**Figure 3:**
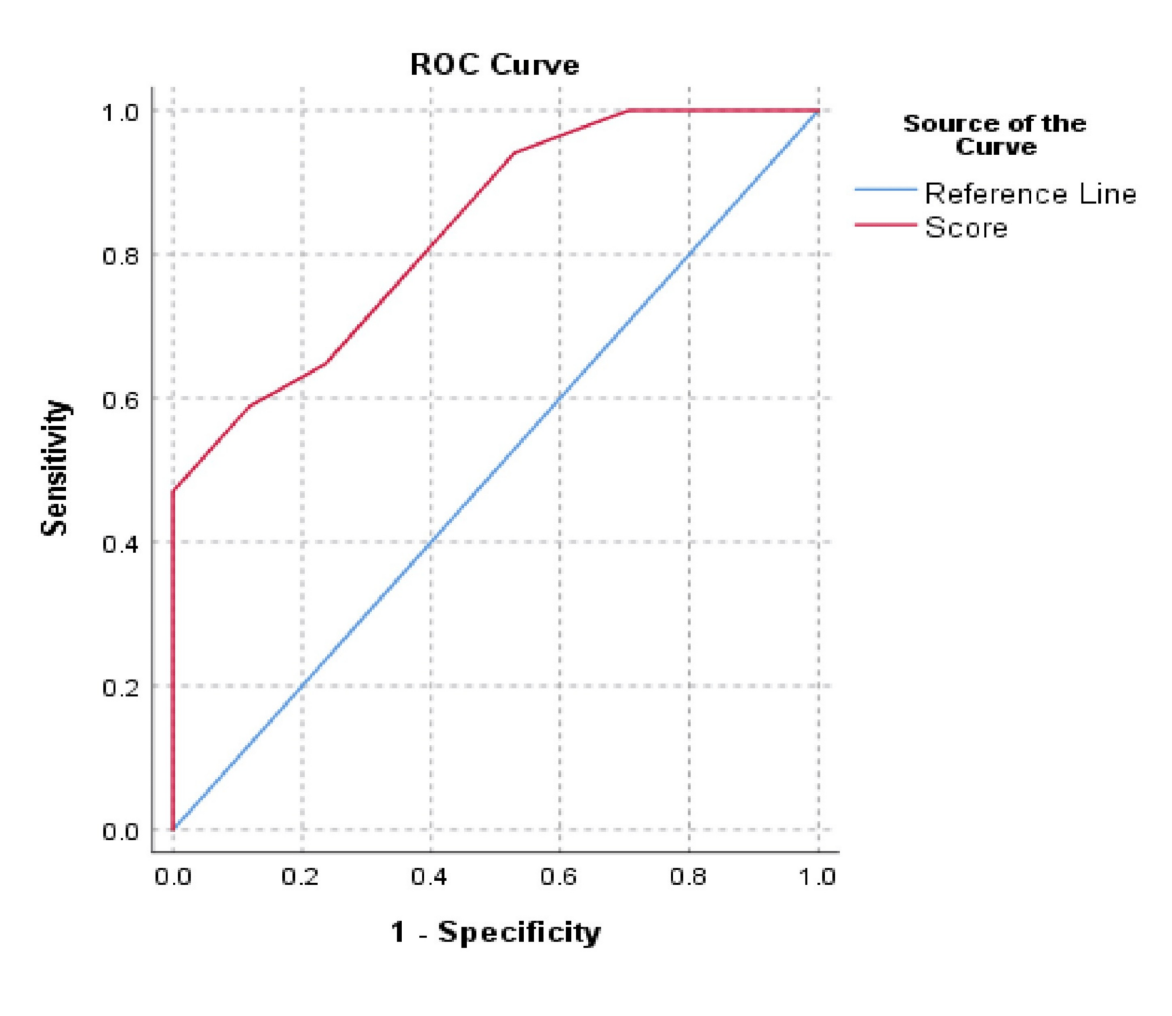
ROC curve for the Sodergren score with respect to surgical treatment AUC c= 0.834 (95% CI 0.702-0.966) ROC: Receiver operating characteristic; AUC: Area under the curve

Receiver operating characteristic (ROC) curve for the Sodergren score was constructed to find out the cut-off value with respect to surgical treatment (Figure 3). The Sodergren score demonstrated a discriminative ability to predict need for surgery among the HD patients with an AUC of 0.834 (95% CI 0.702 - 0.966). The operating characteristics (sensitivity, specificity, positive predictive value, and negative predictive value) were calculated at the thresholds of ≥4, ≥6, and ≥7. These have been presented in Table 3. At a threshold of ≥7, the Youden index was highest (J=0.47), with a sensitivity of 58.8%, specificity of 88.2%, positive predictive value of 88.3% and negative predictive value of 68.2%. The Hosmer and Lemeshow test showed a good calibration of the model with p=0.615.

**Table 3:** Operating characteristics at Sodergren score thresholds of ≥4, ≥6 and ≥7

Operating characteristic	≥4	≥6	≥7
Sensitivity (95% CI)	94.1 (71.3-99.9)	64.7 (38.3-85.8)	58.8 (32.9-81.6)
Specificity (95% CI)	47.1 (22.9-72.2)	76.5 (50.1-93.2)	88.2 (63.6-98.5)
Positive predictive value (95% CI)	64.0 (52.8-73.9)	73.3 (52.1-87.4)	83.3 (56.2-95.1)
Negative predictive value (95% CI)	88.9 (52.8-98.3)	68.4 (51.9-81.3)	68.2 (54.2-79.5)

## Discussion

HD is a common anorectal condition affecting millions of people around the world and significantly alters the quality of life of a substantial number of working-age individuals [12]. A recent International survey evaluated the prevalence of HD in a representative sample of the general population across a number of countries and found the HD prevalence to be 11% [13]. Studies from several countries have reported the prevalence of HD ranging from 14.4% to as high as 38.9% [14-16]. By the age of 50 years, about 50% of the population is estimated to have had HD at some point in their life, and around 5% are estimated to have had symptomatic HD [17-19]. Thus, despite its low morbidity, the high prevalence and high impact on the quality of life of the patient make it important to develop and validate tools to aid clinical decision making, which are centred around the quality of life of the patient. 

Many symptom severity scores have been developed for HD. However, the Sodergren score is the only one that demonstrated the ability to predict the need for surgery in patients. Sodergren score assesses a combination of frequency as well as severity of symptoms to determine the final score. The primary goals of this investigation were to ascertain the difference in Sodergren score among the non-surgical and surgical groups and to evaluate a cut-off score for predicting the need for surgery. With a median score of 7 versus 4 in the surgical and non-surgical groups, respectively (p=0.001), the study found a substantial difference in the scores between the two groups. This differs from the median scores calculated by Sha et al., which were 4 for the surgical group and 0 for the patients undergoing rubber band ligation [10]. The scores in our study are more in line with those found by Pucher et al., which showed a mean score of 7.7±3.9 vs 2.8±3.5 for surgical vs ambulatory/no treatment groups (p=0.002) [9]. Nonetheless, the Sodergren score demonstrated the ability to assign higher scores to patients needing surgery in all these studies.

In our study, the Sodegren score showed a satisfactory ability to predict the need for surgery in HD patients, with an AUC of 0.834. While this result differs from the one found by Sha et al. (AUC=0.735, 95% CI 0.675-0.795), it is strikingly similar to the one found by Pucher et al. (AUC=0.842, 95% CI 0.714-0.970) [9,10]. One of the reasons for such a similarity in results could be due to the smaller sample size in Pucher et al. (n=45), which is similar to our sample size of n=34, while Sha et al. had a much larger sample size of n=290 [9,10].

Based on the operating characteristics calculated at each threshold, the value of ≥7 showed the highest Youden index (0.47), with a sensitivity of 58.8%, specificity of 88.2%, a positive predictive value of 83.3% and a negative predictive value of 68.2%. Thus, we propose a cut-off score of ≥7 for determining the need for surgery. This differs from the findings of Sha et al., who proposed a cut-off of ≥6 with a sensitivity of 44.3%, specificity of 83.2%, positive predictive value of 60.3% and negative predictive value of 72.2% [10]. Pucher et al. did not propose any cut-off value in their study [9]. Our study found a significant difference in the scores of the two arms and also showed a good AUC.

Globally, the integration of PROMs into proctological practice is gaining traction. Several scores, such as the Sodergren score [9], the Hemorrhoid Severity Score [20], and the Hemorrhoidal Disease Symptom Score and Short Health Scale for Hemorrhoidal Disease (HDSS and SHS-HD) [21] have been developed over the recent years. Recent clinical trials in HD have included a PROM as one of their major outcome measures, thus further substantiating the growing use of PROMs for objective measurement of therapeutic efficacy [22,23].

Adoption of PROMs in the Indian clinical settings still remains limited. While our results are promising, further studies with long-term follow-up, utilizing and comparing PROMs such as the Sodergren score, are required to establish their efficacy in the Indian clinical settings for HD. 

Our study had the limitation of being confined to the outpatient setting. There was no follow-up of the patients. No post-treatment score was taken to assess the effect of treatment on the Sodergren score. The study was purely cross-sectional in nature.

## Conclusions

Our cross-sectional study found the Sodergren score to be an effective PROM for guiding clinical decision making in HD. Patients needing surgery reported a significantly higher score as compared to those on conservative therapy. Sodergren score cut-offs could reliably predict the need for surgery in HD patients. Limitation of our study was an outpatient setting and without follow-up. The post-treatment score was not taken to assess the effect of treatment on the Sodergren score. Further studies involving long-term follow-up and comparison of treatment outcomes are essential to establish the use of Sodergren score in routine clinical practice.
